# Artificial Intelligence and Diagnostics in Medicine and Forensic Science

**DOI:** 10.3390/diagnostics13233554

**Published:** 2023-11-28

**Authors:** Thomas Lefèvre, Laurent Tournois

**Affiliations:** 1 IRIS—Institut de Recherche Interdisciplinaire sur les Enjeux Sociaux, USPN EHESS CNRS INSERM, 93300 Aubervilliers, France; 2 BioSilicium, France & Université Paris Cité, CNRS, 75012 Paris, France; laurent.tournois@biosilicium.fr

**Keywords:** artificial intelligence, digital revolution, diagnosis, machine learning, forensics, legal medicine, augmented human, data linkage

## Abstract

Diagnoses in forensic science cover many disciplinary and technical fields, including thanatology and clinical forensic medicine, as well as all the disciplines mobilized by these two major poles: criminalistics, ballistics, anthropology, entomology, genetics, etc. A diagnosis covers three major interrelated concepts: a categorization of pathologies (the diagnosis); a space of signs or symptoms; and the operation that makes it possible to match a set of signs to a category (the diagnostic approach). The generalization of digitization in all sectors of activity—including forensic science, the acculturation of our societies to data and digital devices, and the development of computing, storage, and data analysis capacities—constitutes a favorable context for the increasing adoption of artificial intelligence (AI). AI can intervene in the three terms of diagnosis: in the space of pathological categories, in the space of signs, and finally in the operation of matching between the two spaces. Its intervention can take several forms: it can improve the performance (accuracy, reliability, robustness, speed, etc.) of the diagnostic approach, better define or separate known diagnostic categories, or better associate known signs. But it can also bring new elements, beyond the mere improvement of performance: AI takes advantage of any data (data here extending the concept of symptoms and classic signs, coming either from the five senses of the human observer, amplified or not by technical means, or from complementary examination tools, such as imaging). Through its ability to associate varied and large-volume data sources, but also its ability to uncover unsuspected associations, AI may redefine diagnostic categories, use new signs, and implement new diagnostic approaches. We present in this article how AI is already mobilized in forensic science, according to an approach that focuses primarily on improving current techniques. We also look at the issues related to its generalization, the obstacles to its development and adoption, and the risks related to the use of AI in forensic diagnostics.

## 1. The Diagnostic Approach as a Matching of Two Spaces, That of Pathologies and That of Symptoms

The concept of diagnosis is as old as the idea of pathology itself, of harm affecting a living being, even before being a key element of what would become science and medical practice. It is a category resulting from a partition of the pathological states observed in living beings.

Thus, we can define diagnosis as being based on the following elements:

- The knowledge, the definition of a condition, a pathology;
- The identification of direct or indirect signs making it possible to confirm the diagnosis (positive diagnosis), to exclude others (differential diagnosis), or to affirm a cause for a condition (etiological diagnosis).

Diagnosis differs from prognosis, which is a forecast, generally based on a statistical approach, possibly probabilistic [1].

There are therefore two spaces to be related: that of the categories of conditions (pathologies), and that of the signs (the symptoms). In other words, diagnosis involves an operation between two spaces. One may think of classifications of diseases and their associated criteria (signs, symptoms), such as the DSM-5 (Diagnostic and Statistical Manual) [2] or the ICD-11 (International Classification of Diseases) [3]. Thus formulated, we can quickly translate this definition into the digital world. The different signs observed can be transformed into data, and so can the classification of diseases. It is then possible to imagine various forms of processing of these data with computers. Artificial intelligence (AI) can play a key role in such processing.

AI can intervene in both spaces at the same time, but also (and this is undoubtedly its expected use or the most widespread use currently being developed) on the mapping operation between these two spaces—therefore, on the diagnostic process. It can also act as an interface between data, knowledge, and a human operator.

In medicine, just as in forensic science, there are a variety of diagnoses and a variety of signs that can be observed, either directly by the practitioner or via tools or additional examinations. In forensic medicine more specifically, we may think of the different causes and mechanisms of death, the diagnosis of the postmortem interval, and traumatic lesions in the field of thanatology, or so-called functional diagnoses in the field of clinical forensic medicine, such as traumatic lesions, psychotraumatic reactions, or post-traumatic stress disorder.

## 2. A Few Reminders about AI and Data

Before going further into what AI can bring in terms of diagnostics in forensic science, it seems necessary to recall a few notions concerning digital technology and AI. Why deal with the subject of AI here?

### 2.1. From Expert Systems to Machine Learning—What Has Changed?

Indeed, AI is not a new concept or technique, since its emergence can be traced back to the 1950s and 1960s. AI, in general, has the mission of automating tasks that are usually performed by a human operator—in particular, tasks involving cognitive work. Very early on, there were programs, which were then called expert systems, some of which were dedicated to medical diagnosis, and whose performance was similar to that of doctors in the context of certain pathologies [4]. Nevertheless, these systems were based on the explicit formulation of rules established with one or more experts, and concerned at best a discipline, if not a targeted pathology, and had to describe all the possibilities with which a doctor could be confronted, even those which, in the end, they never had to implement or consider, otherwise the program would not have known how to react and conclude when faced with a new case to diagnose.

We have not used these expert systems in routine practice over the last few decades. Why would AI be more relevant today? The differences between the 1950s and 1960s and today relate not only to the nature of the programs themselves but also, and above all, to the conditions surrounding the possibility of developing and using relevant AIs.

Concerning the programs, we may summarize the situation in a way that is somewhat caricatural but generally true in principle as follows: in the era of expert systems, it was necessary to explicitly describe all the possible combinations of signs in connection with diagnoses—the rules were fixed and explicit. AI has become much more efficient and versatile in its applications ever since we relaxed this constraint and “let the program learn” from the data—the “examples”—that we provide to it. A classic way to model the program is to modify it as it is shown new examples to classify, and it continues to make mistakes while still being able to improve. We thus entered the era of machine learning [5].

This implies that, unlike with expert systems, data are needed in order to create an AI—and generally, an AI is only likely to become successful on the basis of a large, if not very large, volume of examples, thus of data. The proliferation of data, resulting from the digitization of our private and professional activities, is key to the development of AI. You need data for the AI’s learning steps, and data to characterize the new cases to be classified by the AI: you therefore need what is called an information system, into which the AI will be able to fit, to plug in somehow. The widespread generalization of the use and availability of computers over the last thirty years has also made the use of AI on a daily basis more realistic. Finally, two other important characteristics relate to the increase in both data storage and data processing capacities—in other words, the power of computers. It goes without saying that these capacities are incomparable to those existing in the 1950s and 1960s, not to mention the huge increase in the accessibility of computers today.

The development of AI is thus taking place amid an environment that is becoming more favorable to it every day, and in the context of the expansion of computerization on the one hand, and automation on the other, as can be seen in the industrial sector with the generalization of robotics on assembly lines, for example.

### 2.2. AI and Forensic Science

In forensic medicine, AI is mainly used in research projects for thanatology or clinical purposes, especially the determination of the causes of death, the estimation of the postmortem interval (PMI), postmortem and antemortem identification, bruise dating, and the assessment of the risk of reoffending. However, forensic pathologists and forensic physicians do not currently seem to use AI in their practice [6]. The lack of data and the potential presence of biases and imbalanced data in AI-based model development projects may prevent the integration of AI in expert assessments. Indeed, AI models are mainly designed using machine learning techniques that require large amounts of data, such as artificial neural networks [7]. Thus, AI models designed on too little or poor-quality data, such as partial data with a lack of features or misannotated data, may lead to non-usable models. Moreover, models designed on imbalanced data may not be generalizable on new data. In other words, such models are not applicable for data collected in real settings [8]. Model applicability also depends on data representativeness. Indeed, AI models developed and tested on non-representative data from a given application may not perform well on data used in real settings for that application. For instance, an AI model designed to estimate the age of an individual and tested on people aged up to 20 years may not be applicable to people aged over 20. That is why data analysis prior to AI development is critical in order to identify and resolve any data-related issues. Therefore, data may be considered as the core of any AI model.

## 3. AI and Disease Categories: Redefining, Discovering, and Stratifying

AI can help to separate and better define pathologies, at least in theory: this was the subject of an ambitious project concerning mental pathologies led by the NIMH (National Institute of Mental Health) in the United States. The idea was to collect data of various kinds, concerning clinical practice, the signs reported by people, imaging, genetics, etc., and to use modern data analysis tools to bring out clear bases for each pathology, or even revise the current classification and discover other pathologies or sub-categories. However, its main designer has had to acknowledge its lack of success thus far [9].

Currently, there is a particular interest in very high-dimensional biological data, such as genetic sequencing data, and, more generally, “omics” data. These data are extremely voluminous, almost impossible for humans to directly interpret, and are therefore a prime target for exploration using AI.

In forensic science, we can thus expect AI to help us better separate certain categories, either through a more efficient analysis of the data or through the enrichment of external data, for example, to better discriminate between accident, suicide, and homicide as circumstances of death. AI also offers the possibility of carrying out multi-category analyses, and not just binary ones (for example, choosing between just two causes of death): in the event of several concurrent mechanisms for death (an acute or pre-existing pathological state, toxicology data, genetic predispositions, functional diagnoses, etc.), it can propose a result that takes into account all the concurrent mechanisms leading to death.

We may also mention the use of data that are “external” to people, for example from connected objects.

The use of external personal data from wearable objects, such as smartwatches, that monitor and record health parameters may be an interesting lead to establish a physio-pathological trajectory before death. Although the use of AI from IoT (Internet of Things) data to diagnose or predict health-related events is currently thriving [10], such device application in forensic medicine diagnosis is poorly reported in the literature. However, the use of such data in forensic practice might help the forensic practitioner to determine the causes of death. For instance, the forensic pathologist might retrieve the pacemaker data from a deceased person to determine or exclude causes of death as well as to estimate the PMI [11]. Data from connected biosensors such as oximeters [12], blood glucometers [13], or electrochemical sensors [14] might also be exploited in the future as complementary elements to determine the cause of death. Moreover, observational data from non-medical people may be used for forensic diagnosis. For example, Jeblee et al. developed machine learning models to determine the cause of death from verbal autopsy narratives, that is to say, interviews with the family members of the deceased person about their health state. The best model reached a precision of 0.773 and a sensitivity and F1-score of 0.77 for 15 categories of causes of death and for people aged between 15 and 69 years [15]. Obviously, validation studies must be carried out before any use of external data in forensic practice.

### 3.1. Some Examples of Expected, Classic Uses of AI in Forensic Science

The common uses of AI in forensic diagnosis mainly concern the assessment of a diagnosis, the severity of a pathology or an injury, and the stratification of risks. Indeed, AI-based classification models may compute the probability of presenting a given diagnosis or belonging to a given category, for a given case or person. Therefore, classification models may provide a probability for given diagnoses with a given pathology. For instance, Lin et al. used neural networks to diagnose five causes of death from spectrochemical analyses of pulmonary edema fluid with a mean accuracy above 90% [16]. Zeng et al. used convolutional neural networks to diagnose fatal hypothermia from PMCT (postmortem computed tomography) images with a best area under the ROC curve of 0.956 [17]. Schweitzer and Thali used neural networks to diagnose fatal obstructive asphyxia from pulmonary PMCT with a correct recognition rate above 95% based on few cases [18]. Moreover, the characteristics of an injury may be determined through AI-based models. For instance, in forensic anthropology, Dempsey et al. used machine learning models to determine if a femur bone wound was due to a vertical impact (a fall) or a horizontal impact (vehicle impact) based on femur radiographs. The final model achieved an average precision, recall, and F1-score of 0.81, 0.71, and 0.66, respectively, from a dataset of 103 cases [19]. AI-based models are also able to determine or assess the severity of an injury or a pathology. For example, Garland et al. examined fatal head injury identification using convolutional neural networks from PMCT images. Through this feasibility study, the authors achieved an accuracy of 0.7 with little data [20]. In forensic anthropology, Demir et al. developed machine learning models to categorize humerus fractures according to the number of pieces produced by the underlying trauma based on shoulder and arm radiographs. The best model showed an accuracy of 0.9912 [21]. Finally, the stratification of risks is mainly applied in forensic psychiatry to assess the risk of recidivism [22] or violence [23]. However, the use of AI in this forensic medicine field is still controversial due to potential model biases [24]. Therefore, further model developments are required before application in the justice system.

### 3.2. AI beyond the Mere Improvement of Pre-Existing Techniques: The Example of Postmortem Interval Estimation

One of the main advantages of AI is the use of multiple data types for a given application. Indeed, AI models may be designed from any type of numerical data, such as continuous or discrete variables, photos, videos, or texts, and are able to combine those data types to improve model performance and reliability [25]. For instance, the methods used in PMI estimation are based on taphonomic processes; biomolecular, environmental, and entomological analyses; and postmortem imagery [26]. All those fields of expertise may be leveraged using AI to improve PMI estimation. In any case, AI is already used in research projects to help experts with PMI estimation based on eye opacity [27], electrolyte concentration in the vitreous humor [28], the body’s microbiome [29], insect identification [30], and multiomics approaches [31]. However, one may point out that those sources of data are specific to a field of expertise, which may explain why there is currently no AI model that integrates all those data to estimate PMI, since the complexity of each field of expertise would require a large-scale collaboration project between several laboratories or institutes. Nevertheless, such a project would represent an opportunity for improvements in data storage and sharing in forensics. Therefore, AI might also enhance forensic research through the developments required to design AI-based models from multiple fields of expertise.

## 4. AI and Symptoms: From Signs to Data

Classical medicine was based on the collection of physical signs that were essentially accessible to the human senses: touch/palpation, hearing/percussion, taste, sight, smell. Then, humans equipped themselves with tools allowing them either to increase their sensory capacities (stethoscopes) or to add dimensions/extend the spectrum of observation, for example through complementary examinations (biology, imaging, microscopes, etc.).

Diagnoses are essentially based on the following elements: (i) what is accessible as signs, therefore depending on human and technical means; (ii) an experimental method making it possible to test the links between signs and pathology; and (iii) a cultural context that contributes to defining what is pathological and what is normal.

- I—The set of accessible signs has been enriched with digitization: there are of course all the existing signs identified to date, which will be enriched by the practical and individual consideration of signs not used so far: either new signs identified in the masses of information available today, or the use of known but non-clinical signs or signs from complementary examinations—for example, signs from epidemiology, such as social position. The limit is no longer the human limit of the doctor or additional examinations, but of everything that can be digitized and linked to a patient.
- II—The search for causality is at the foundation of medicine, at least in its interventionist dimension: we seek to establish a robust link between exposure and pathology. The observable signs can be signs causally linked to the pathology (expression of the pathology, but rarely pathognomonic), or associated signs, in the sense, for example, of markers of a pathology (such as risk factors or correlations). In fact, digitization and the use of AI extend the approach of linking signs and pathologies, with, in particular, at least two possibilities that are difficult to access using conventional statistics: the identification of non-linear associations, and the identification of causality networks. Moreover, this causality or multicausality can even become calculable and “individualizable” (even if it remains of probabilistic expression) with each new case that is presented.
- III—Although international classifications are increasingly used to ensure a minimum level of reproducibility in research and a common language throughout the world, the notion of what is pathological or normal remains imbued with cultural values. An additional step in the search for a certain reproducibility and objectivity in diagnosis could involve a massive increase in the use of data and AI, which are more capable of delimiting pathological or healthy criteria on the basis of many criteria. On the other hand, we must beware of the biases of representativeness of cultural norms and populations that are very present today in the data, and that AI can reproduce or even reinforce.
- Thus, in forensic science, as in medicine, the use of data, new sensors or additional examinations, and new analysis methods such as AI can both extend and modify known diagnoses as well as diagnostic approaches. The key difference is that AI is based on data, while doctors base their work on signs. The correspondence between data and signs is not obvious and is a construction that must be mastered and controlled.

## 5. AI as an Operation between Signs and Pathology, as a Diagnostic Operation

AI feeds on data to produce a result—here, a categorization, a diagnosis.

While we barely touch on the space of signs and not at all on the space of pathologies, one of the hopes and applications of AI today lies in an improved diagnostic performance, according to several possibilities: better precision, greater speed, better accessibility, better reliability, etc., compared to current techniques or compared to humans, either alone or with the help of AI.

### 5.1. Some Examples in Forensic Science Diagnoses: Applications and Performance

In forensic diagnosis, AI already outperforms human experts or gold standard methods (see Table 1). For instance, Zhou et al. developed an AI-based diatom detection model for the diagnosis of drowning from whole-slide sample images, which is about twice as fast as human experts and with a similar detection accuracy (AUC = 0.9951). Compared to the gold standard method, this model is able to handle about 60% of the human work [32]. In a later study, the same authors developed DiatomNet, a diatom detector trained on images containing impurities, and reached a precision, recall, F1-score, and area under the precision–recall curve of 0.969, 0.968, 0.968, and 0.991, respectively [33]. AI may also perform better in the determination of the cause of death than standard software. For example, Falissard et al. designed a neural network that is able to determine the cause of death from death certificates better than the Iris software [34]. According to the performance results given by the authors, this neural network outperformed the software by more than 0.2 accuracy points. Therefore, AI may also be able to perform better than state-of-the-art software. In forensic anthropology, Byeon et al. designed a neural network to discriminate cut marks from trampling marks on bone images. On 20 experimental marks, the neural network achieved an average accuracy of 0.91 and an average sensitivity and specificity of 0.9, whereas the average accuracy of taphonomy experts reached 0.63 [35]. In forensic ballistics, Savakar and Kannur developed an AI-based ensemble classifier for weapon type identification based on skin wound features. According to the authors, the proposed model’s accuracy ranges from 0.97 to 0.99, whereas the traditional method’s accuracy ranges from 0.75 to 0.92, which makes the AI-based model more accurate than the traditional method [36].

These results demonstrate that AI may help forensic experts with very specific tasks. Indeed, in the examples mentioned above, AI models perform better than human experts or standard methods. Moreover, these models are able to process large amounts of data faster than humans. However, they may be subject to biases or may not be applicable in real settings due to small and non-representative training data. Therefore, human expertise is still required in the overall decision process of a forensic diagnosis.

While AI can perform as well or better than humans in diagnosis, there may be a specific limit to these models with regard to their adoption by doctors: doctors trust their reasoning, which they can explain to another human, whereas most AIs will not be able to “explain” to a human in an understandable way why they achieved such and such a result. This is the problem of explainability.

### 5.2. Explainability or Explainable AI and the Problem of Diagnosis

In forensic medicine, current AI models mainly rely on deep learning techniques, which are opaque to human interpretation. Such models are often referred to as “black-box models”. An entire research field called explainable artificial intelligence (XAI) aims at handling this issue. The main objective of XAI techniques is to clarify the decision process of a given AI model for human understanding. Nowadays, XAI is able to provide elements of model explainability through the determination of features’ importance for variable-based models or pixels’ importance for image-based models in forensic medicine. For instance, in forensic diatomology, Zhou et al. used an XAI technique called Grad-CAM (gradient-weighted class activation mapping) to highlight the areas that the neural network focused on to detect a diatom from a sample slide image for the diagnosis of drowning [32]. In forensic entomology, Apasrawirote et al. used heatmaps of attention maps to highlight the image areas of posterior spiracles from fly maggots on which the neural network mainly relies to identify fly species for PMI estimation. The authors also extracted and visualized the patterns analyzed with the network to identify four fly species [37]. In forensic anthropology, Cifuentes-Alcobendas and Domínguez-Rodrigo used Grad-CAM to identify the cut mark image areas on which the neural networks focus to determine if the cut was performed on fleshed or de-fleshed bone [38]. These visualization techniques make it possible to identify potential biases or spurious correlations in the decision process of convolutional neural networks and provide elements of model explainability for the human user. Therefore, in forensic diagnosis, XAI techniques enhance AI transparency and trustworthiness. However, current XAI applications provide elements of interpretation rather than a thorough human-readable decision process in forensic diagnosis. Indeed, after highlighting the regions of importance on an image, how does the neural network interpret those regions of interest (in a human-understandable way) to make a prediction? No study has yet been performed to answer this question in forensic medicine. Therefore, AI models cannot currently replace human experts in forensic diagnosis. Nevertheless, these AI models may be used to help forensic experts in a specific task and provide decision-making elements for a given expert assessment. As a consequence, AI should be considered as a virtual assistant rather than a full-fledged skilled expert in forensic diagnosis.

## 6. AI as an Assistant

We tend to imagine the place of AI in relation to humans at one of two extremes: either in a weak position, almost invisible and indistinguishable from the other tools available to humans, as one more or less sophisticated software component among others; or conversely as a substitute for humans. The rise of AI confirms its relevance in the first case, but it also brings about an intermediate position, as an assistant to human beings. This assistance can start in the simple form of “classic” diagnostic decision support software; for example: ultimately, the practitioner is unable to detect, based on the results provided, whether it was an AI or a less evolved algorithm that performed the task. In particular, the interactions with the software do not differ from those that one can have in computing. However, the real novelty lies in both the nature of the interactions between machine and human, but also the complexity and diversity of the tasks that can be asked of an AI.

### 6.1. AI and the Emergence of More “Human” Interactions with Machines

Computing has accustomed us to using pointers, like the mouse, to select an item or act through a graphical interface with a computer. The advent of smartphones in particular then accustomed us to touching interfaces: these do not replace pointers with the same ergonomics or ease, but rather are complementary and work very well in many examples of simplified uses. Even more recently, advances in augmented reality or virtual reality (mixed reality) [39] have introduced other ways of interacting with a computer or its environment, which is made possible or more efficient thanks to both the development of hardware components (notably cameras) but also AI-type calculation and processing capabilities for real-time pattern recognition: you can now navigate with your eyes (eye-tracking) or with your (empty) hands. Finally, voice recognition technology and, conversely, voice synthesis such as text-to-speech (an artificial voice can read written text) also brings other ways of interacting with a machine—also facilitated by more powerful AI.

Since the end of 2022, we have been witnessing the emergence of particularly efficient chatbots in terms of the quality of production of credible texts, correctly written, in real time, and which can give rise to interesting human/machine dialogues. These chatbots, like ChatGPT [40], have become possible thanks to an important triptych: the performance of the language models on which they are based (large language models, or LLM, like GPT-4); the development of a technique for taking context into account; and finally, significant human involvement in selecting the most likely and acceptable responses from a human perspective. It is important to remember that at this time, through construction and by nature, these AIs are intended to produce texts that are credible and have a human conversational look, by means of a mechanism of the maximum likelihood of the next word, and they do not require any reasoning or aim to establish a true or even real result. It is a pure conversation simulation program. Nevertheless, this improved performance makes the prospect of more fluid and natural interactions between machine and human much more accessible and realistic.

### 6.2. “Invisible” AI to Help with Machine/Machine Dialogue

Finally, there is a dimension of the use of AI which will increase in the years to come and which is not necessarily visible to us on a daily basis: that which allows us to interact with or to help program other machines, to create what is called machine-to-machine—machines or software dialoguing with other machines or software. AIs already make it possible to simplify or improve the speed and reliability of certain intermediate operations in certain tasks. Thus, we can use AI to gather sparse data, “structure” it (put it in the same format, in a table), in order to make it compatible with another standard necessary for the use of a given software, for example. This is typically what we have implemented to facilitate multicenter research in forensic medicine in France, with the ORFeAD network [41]. To put it briefly, ORFeAD makes it possible to federate the data resulting from the activity of 12 French forensic centers, and to structure them without prior homogenization or specific tasks in each of the centers. The centers send the medical files to ORFeAD, which, via different forms of AI processing, including natural language processing, extracts the information sought and puts it into data that can be used by each participating center.

### 6.3. AI as an Assistant to Medical Reasoning

Finally, AI has for several years now been able to help model a certain type of reasoning, no longer by asking experts to explain all the possible decision-making rules, including those they have never come across in their practice, but either solely from the data or in a hybrid way, i.e., from the data and from indications from the expert. These hybrid approaches comprise, in particular, all the techniques based on Bayesian networks, which have been introduced into forensic medicine in different forms and via different complementary approaches: they range from the modeling and quantification of conditional probabilities in medical reasoning [42], to the representation of multicausal networks, which also enable the calculation of conditional probabilities, based on data [43]. The advantage of these approaches is that they rely on an explicit graphical representations, which a human can read and explain or control.

## 7. Perspectives and Conclusions

To date, the concrete experience that we have of AI is either in the field of privacy (automatic recognition of photos on a smartphone, for example), or in the form of research in the medical professional field, with applications authorized in particular by the FDA which are beginning to arrive on the market, the vast majority of which relate to diagnostic support applications in imaging.

Five years ago, the role of AI in medicine was almost exclusively a matter of research. Today, we are beginning to see its applications intended for everyday practice, which are highly targeted. We are therefore entering a period where we will be able to assess, in real life, the perceived and real contribution of AI to everyday practice, but also its acceptability and the adaptations it undergoes in the hands of practitioners. Obviously, the requirements existing in classical medicine, reliability and precision in particular, are doubled in forensic medicine, since the use of AI for diagnostic purposes must be accepted by the medical community but also within the courts. We are therefore only at the beginning, but the story seems to be going in the direction of a more or less widespread, relatively natural adoption of AI. Not only has the environment become propitious to the adoption of AI, but it would seem to make little sense, given the current and constant knowledge in terms of diagnoses and signs, to want to go without potentially more powerful tools than those we currently have.

Nevertheless, the success of AI depends on several factors, which we often take for granted today, though the vast majority of them often remain problematic on a daily basis. The routine use of AI depends on the degree of digitization of medical practices; indeed, the existence of data and information systems is necessary both for the development of AI models and for their integration with existing information systems. AI is also highly dependent on the effective interoperability of different data sources and information systems: in fact, we cannot exclude that in order to function and improve its performance; AI requires the interconnection of separate sources, which do not usually dialogue with each other. The querying of such separate databases, for example, medical data on the one hand and judicial data on the other, of course also raises regulatory questions that ought to be clarified. For example, in France today, it is not legal to interconnect personal data with criminal offense data.

The place of humans in the field of intervention with AI still needs to be clarified, particularly in the medical field, which is both highly regulated and steeped in corporatism. One could consider that it is, above all, a question of the choice of the human actors concerned (here, doctors) to accept or reject the use of such and such a tool in their practice. The counterpart on the criminal justice side presents the same configuration. Beyond the acceptance or not of the tool, the question of responsibility remains: in the short term, there is no prospect of a diagnosis being proposed or even entirely determined using an AI without being certified by a doctor, who will therefore bear the responsibility.

Nevertheless, we cannot ignore at least two key elements that determine professional organizations’ permeability to AI. The first relates to costs and limited qualified resources, particularly in a situation of shortage. A common reaction to a lack of doctors to meet the demand is to try to find alternative solutions, rather than to try to boost the workforce by increasing the volume of trained staff or by improving attractiveness. The promise, realistic or not, of AI in terms of the rapid, reliable, and massive treatment of cases that are usually assigned to doctors is inevitably tempting in such a context. Furthermore, to date, while certain initial training courses in medicine include modules in digital health, these still appear to be too timid or overly abstract and conceptual, while their teaching ought to be concrete, making it possible to measure the interest and limits of AI tools in situ. It is clearly necessary that professionals be trained in AI if they are to be able to use these tools correctly and confidently in a critical manner.

The second element, linked to the first, relates to the evolution of the mode of management and of organizations, and to the decision-making power of the hierarchy in terms of financing and human resources. Again, AI promises to be a cheaper and more profitable investment than a human being. Yet, to date, this appears to be a false promise: equipping oneself with AI entails the existence or the creation of dedicated human resources for the use of the AI, and moreover, it makes the organization captive to the supplier of the AI, whose prices may go up further down the line. The rule in the digital world is that of the colonization of territories devoid of technology. Such clients are initially offered this technology free of charge, in order to make them dependent on it, before being hit with more or less arbitrary tariffs, depending on the goodwill of the provider and the degree of captivity of the client. In the end, the choice ultimately falls only very indirectly, if at all, to the professionals directly concerned.

For the moment, the few studies comparing the performance of AI and that of experts, essentially in the field of decision support based on image analysis, point to conclusions that may seem logical: AI is incomparably more efficient in terms of processing speed, and its precision is comparable to that of experts, and sometimes even better, but it is the intelligent combination of the two—AI and experts—that gives the best results, exceeding the gold standard.

However, AI seems to be moving, on some fronts, faster than one would have expected even five years ago. The impressive progress of so-called generative AI such as ChatGPT—although it needs to be relativized—portends major upheavals in many fields and professions, which are difficult to predict precisely today. It is probably through this means—new conversational agents and large language models like ChatGPT—which is developing far quicker than was anticipated but which is still insufficiently controlled, that new perspectives, different from those previously imagined or currently being developed, may emerge. It is these tools, called generative AI, which are most likely, through their versatility but also their accessibility, to modify our relationship with AI in the professional environment: primarily through the way in which we interact with it. Interfaces risk being disrupted: it will no longer be a matter of writing or checking boxes on a computer, but of asking questions as one would to a colleague.

Finally, the fact remains that, to date, AI remains massively dependent on humans and must also be assessed by the latter. On the one hand, high-performance AIs are so because they have been fed with data produced via mass human activity, but also, and above all, because in the learning process, humans are just as massively mobilized to correct them, refine them, etc. There is no AI without data, but also no AI without humans yet. On the other hand, since AI feeds on the data we produce, we should not expect an authentically original production coming out of what we give it, and we must keep in mind that it reproduces the biases resulting from our human activities. This is a major limitation, as it can not only give false assurance in the reliability of the AI—there are many things it does not “know” because it is not available in the data, for example, the specificities of heart disease linked to gender and ethnicity—but this can also lead to reinforcing these biases, promoting them as the norm.

AI can be a great tool, which today cannot function without humans. Yet, it is urgent that the professionals concerned by its use be aware of its limits, but also be able to choose how they situate themselves in relation to AI.

## Figures and Tables

**Table 1:** Comparison of performance between AI models and standard methods in forensic medicine diagnoses.

Reference	Performance of the Standard Method	Performance of the AI Model	Main Advantage of the AI Method
Zhou et al. [32]	About 2.5 diatoms identified per minute	About 4 diatoms identified per minute	Improvement in processing speed
Falissard et al. [34]	Accuracy ranges between 0.74 and 0.75	Accuracy ranges between 0.977 and 0.979	Improvement in accuracy
Byeon et al. [35]	Average accuracy is 0.63	Average accuracy is 0.91	Improvement in accuracy
Savakar and Kannur [36]	Accuracy ranges between 0.75 and 0.92	Accuracy ranges between 0.97 and 0.99	Improvement in accuracy

## Data Availability

Not applicable.

## References

[1] Balogh E.P., Miller B.T., Ball J.R., Committee on Diagnostic Error in Health Care, Board on Health Care Services, Institute of Medicine, The National Academies of Sciences, Engineering, and Medicine (2015). The Diagnostic Process. Improving Diagnosis in Health Care.

[2] American Psychiatric Association (APA) (2013). Diagnostic and Statistical Manual DSM 5.

[3] World Health Organization (WHO) (2019). International Classification of Diseases ICD-11.

[4] Shortliffe E.H., Buchanan B.G. (1975). A model of inexact reasoning in medicine. Math. Biosci..

[5] What Is Machine Learning (ML)? Datascience@berkeley, the Online Master of Information and Data Science from UC Berkeley. https://ischoolonline.berkeley.edu/blog/what-is-machine-learning/.

[6] Tournois L., Lefèvre T., Lidströmer N., Ashrafian H. (2021). AI in forensic medicine for the practicing doctor. Artificial Intelligence in Medicine.

[7] Géron A. (2017). Hands-on Machine Learning with Scikit-Learn and TensorFlow: Concepts, Tools, and Techniques to Build Intelligent Systems.

[8] Johnson J.M., Khoshgoftaar T.M. (2019). Survey on deep learning with class imbalance. J. Big Data.

[9] Cuthbert B.N., Insel T.R. (2013). Toward the future of psychiatric diagnosis: The seven pillars of RDoC. BMC Med..

[10] Pradhan B., Bhattacharyya S., Pal K. (2021). IoT-based applications in healthcare devices. J. Healthc. Eng..

[11] Dobson R. (2003). Pacemaker pinpoints time of death in murder case. BMJ.

[12] Kinoshita H., Tanaka N., Takakura A., Jamal M., Ito A., Kumihashi M., Tstsui K., Matsubara S., Kimura S., Ameno K. (2018). Application of CO-Oximeter for Forensic Samples.

[13] DeJoseph M., Hoppa E. (2016). Death investigation of diabetes mellitus: Scene investigation and interrogation of technology. Acad. Forensic Pathol..

[14] Teymourian H., Parilla M., Sempionato J.R., Montiel N.F., Barfidokht A., Van Echelpoel R., De Wael K., Wang J. (2020). Wearable electrochemical sensors for the monitoring and screening of drugs. ACS Sens..

[15] Jeblee S., Gomes M., Jha P., Rudzicz F., Hirst G. (2019). Automatically determining cause of death from verbal autopsy narratives. BMC Med. Inform. Decis. Mak..

[16] Lin H., Luo Y., Sun Q., Deng K., Chen Y., Wang Z., Huang P. (2020). Determination of causes of death via spectrochemical analysis of forensic autopsies-based pulmonary edema fluid samples with deep learning algorithm. J. Biophotonics.

[17] Zeng Y., Zhang X., Yoshizumi I., Zhang Z., Mizuno T., Sakamoto S., Kawasumi Y., Usui A., Ichiji K., Bukovsky I. (2023). Deep learning-based diagnosis of fatal hypothermia using post-mortem computed tomography. Tohoku J. Exp. Med..

[18] Schweitzer W., Thali M. (2019). Fatal obstructive asphyxia: Trans-pulmonary density gradient characteristic as relevant identifier in postmortem CT. J. Forensic Radiol. Imaging.

[19] Dempsey N., Bassed R., Blau S. (2021). The issues and complexities of establishing methodologies to differentiate between vertical and horizontal impact mechanisms in the analysis of skeletal trauma: An introductory femoral test. Forensic Sci. Int..

[20] Garland J., Ondruschka B., Stables S., Morrow P., Kesha K., Glenn C., Tse R. (2020). Identifying fatal head injuries on postmortem computed tomography using convolutional neural network/deep learning: A feasibility study. J. Forensic Sci..

[21] Demir S., Key S., Tuncer T., Dogan S. (2020). An exemplar pyramid feature extraction based humerus fracture classification method. Med. Hypotheses.

[22] Tortora L., Meynen G., Bijlsma J., Tronci E., Ferracuti S. (2020). Neuroprediction and A.I. in forensic psychiatry and criminal justice: A neurolaw perspective. Front. Psychol..

[23] Cockerill R.G. (2020). Ethics implications of the use of artificial intelligence in violence risk assessment. J. Am. Acad. Psychiatry Law.

[24] Lefèvre T., Lidströmer N., Ashrafianin H. (2022). Artificial intelligence in forensic medicine. Artificial Intelligence Medicine.

[25] Qin Z., Zhao P., Zhuang T., Deng F., Ding Y., Chen D. (2023). A survey of identity recognition via data fusion and feature learning. Inf. Fusion.

[26] Sharma R., Diksha Bhute A.R., Bastia B.K. (2022). Application of artificial intelligence and machine learning technology for the prediction of postmortem interval: A systematic review of preclinical and clinical studies. Forensic Sci. Int..

[27] Cantürk İ., Özyılmaz L. (2018). A computational approach to estimate postmortem interval using opacity development of eye for human subjects. Comput. Biol. Med..

[28] Risoluti R., Canepari S., Frati P., Fineschi V., Materazzi S. (2019). “2 n analytical platform” to update procedures in thanatochemistry: Estimation of post mortem interval in vitreous humor. Anal. Chem..

[29] Liu R., Gu Y., Shen M., Li H., Zhang K., Wang Q., Wei X., Zhang H., Wu D., Yu K. (2020). Predicting postmortem interval based on microbial community sequences and machine learning algorithms. Environ. Microbiol..

[30] Beyramysoltan S., Ventura M.I., Rosati J.Y., Giffen-Lemieux J.E., Musah R.A. (2020). Identification of the species constituents of maggot populations feeding on decomposing remains-Facilitation of the determination of post mortem interval and time since tissue infestation through application of machine learning and direct analysis in real time-mass spectrometry. Anal. Chem..

[31] Bonicelli A., Mickleburgh H.L., Chighine A., Locci E., Wescott D.J., Procopio N. (2022). The “ForensOMICS” approach for postmortem interval estimation from human bone by integrating metabolomics, lipidomics, and proteomics. eLife.

[32] Zhou Y., Zhang J., Huang J., Deng K., Zhang J., Qin Z., Wang Z., Zhang X., Tuo Y., Chen L. (2019). Digital whole-slide image analysis for automated diatom test in forensic cases of drowning using a convolutional neural network algorithm. Forensic Sci. Int..

[33] Zhang J., Vieira D.N., Cheng Q., Zhu Y., Deng K., Zhang J., Qin Z., Sun Q., Zhang T., Ma K. (2023). DiatomNet v1.0: A novel approach for automatic diatom testing for drowning diagnosis in forensically biomedical application. Comput. Methods Programs Biomed..

[34] Falissard L., Morgand C., Roussel S., Imbaud C., Ghosn W., Bounebache K., Rey G. (2020). A deep artificial neural network-based model for prediction of underlying cause of death from death certificates: Algorithm development and validation. JMIR Med. Inform..

[35] Byeon W., Domínguez-Rodrigo M., Arampatzis G., Baquedano E., Yravedra J., Maté-González M.A., Koumoutsakos P. (2019). Automated identification and deep classification of cut marks on bones and its paleoanthropological implications. J. Comput. Sci..

[36] Savakar D., Kannur A. (2018). Ensemble learning approach for weapon recognition using images of wound patterns: A forensic perspective. Int. J. Image Graph. Signal Process..

[37] Apasrawirote D., Boonchai P., Muneesawang P., Nakhonkam W., Bunchu N. (2022). Assessment of deep convolutional neural network models for species identification of forensically-important fly maggots based on images of posterior spiracles. Sci. Rep..

[38] Cifuentes-Alcobendas G., Domínguez-Rodrigo M. (2019). Deep learning and taphonomy: High accuracy in the classification of cut marks made on fleshed and defleshed bones using convolutional neural networks. Sci. Rep..

[39] Smith R.T., Clarke T.J., Mayer W., Cunningham A., Matthews B., Zucco J.E. (2020). Mixed reality interaction and presentation techniques for medical visualisations. Adv. Exp. Med. Biol..

[40] OpenAI’s ChatGPT. https://openai.com/blog/chatgpt.

[41] Outils et Reseau pour la Fédération, l’utilisation et l’analyse de Données en Médecine Légale ORFeAD. https://orfead.org/.

[42] Aitken C., Mavridis D. (2019). Reasoning under uncertainty. Evid. Based Ment. Health.

[43] Lefèvre T., Lepresle A., Chariot P. (2015). Detangling complex relationships in forensic data: Principles and use of causal networks and their application to clinical forensic science. Int. J. Leg. Med..

